# Microvascular cerebral blood flow response to intrathecal nicardipine is associated with delayed cerebral ischemia

**DOI:** 10.3389/fneur.2023.1052232

**Published:** 2023-03-17

**Authors:** Eashani Sathialingam, Kyle R. Cowdrick, Amanda Y. Liew, Zhou Fang, Seung Yup Lee, Courtney E. McCracken, Feras Akbik, Owen B. Samuels, Prem Kandiah, Ofer Sadan, Erin M. Buckley

**Affiliations:** ^1^Wallace H. Coulter Department of Biomedical Engineering, Georgia Institute of Technology, Emory University, Atlanta, GA, United States; ^2^Department of Electrical and Computer Engineering, Kennesaw State University, Marietta, GA, United States; ^3^Center for Research and Evaluation, Kaiser Permanente Georgia, Atlanta, GA, United States; ^4^Division of Neurocritical Care, Department of Neurology and Neurosurgery, Emory University School of Medicine, Atlanta, GA, United States; ^5^Department of Pediatrics, School of Medicine, Emory University, Atlanta, GA, United States; ^6^Children's Research Scholar, Children's Healthcare of Atlanta, Atlanta, GA, United States

**Keywords:** vasospasm, diffuse correlation spectroscopy, nicardipine, blood flow, subarachnoid hemorrhage

## Abstract

One of the common complications of non-traumatic subarachnoid hemorrhage (SAH) is delayed cerebral ischemia (DCI). Intrathecal (IT) administration of nicardipine, a calcium channel blocker (CCB), upon detection of large-artery cerebral vasospasm holds promise as a treatment that reduces the incidence of DCI. In this observational study, we prospectively employed a non-invasive optical modality called diffuse correlation spectroscopy (DCS) to quantify the acute microvascular cerebral blood flow (CBF) response to IT nicardipine (up to 90 min) in 20 patients with medium-high grade non-traumatic SAH. On average, CBF increased significantly with time post-administration. However, the CBF response was heterogeneous across subjects. A latent class mixture model was able to classify 19 out of 20 patients into two distinct classes of CBF response: patients in Class 1 (*n* = 6) showed no significant change in CBF, while patients in Class 2 (*n* = 13) showed a pronounced increase in CBF in response to nicardipine. The incidence of DCI was 5 out of 6 in Class 1 and 1 out of 13 in Class 2 (*p* < 0.001). These results suggest that the acute (<90 min) DCS-measured CBF response to IT nicardipine is associated with intermediate-term (up to 3 weeks) development of DCI.

## 1. Introduction

Subarachnoid hemorrhage (SAH) is an uncommon cause of stroke that disproportionately contributes to increased mortality and morbidity. Globally, SAH affects ~8:100,000 people annually, with a mortality rate of ~30% (1, 2). One of the common complications of non-traumatic SAH is delayed cerebral ischemia (DCI) which can occur as early as post-bleed day 3, with the most frequent incidence occurring within 2 weeks (3). DCI affects up to 30% of patients with SAH and leaves them with a reduced quality of life due to motor deficits and/or cognitive dysfunction (4). The pathophysiology of DCI is complex, but it often occurs in the setting of vasospasm, i.e., aberrant constriction of the cerebral vasculature. Vasospasm is a maladaptive response that can contribute to a decrease in cerebral blood flow (CBF) (5, 6). In turn, reduced CBF is associated with spreading cortical depolarization (7) and DCI (8). As ischemic conditions can lead to poor functional outcomes, treating cerebral vasospasm and avoiding DCI after SAH remain a major unmet clinical need.

Calcium channel blockers (CCBs), which act on smooth muscle cells to dilate the vasculature, have been long investigated for their potential role in the treatment of large-artery vasospasm and the prevention of DCI. Indeed, the only guideline-recommended treatment for the prevention of DCI is the oral administration of dihydropyridine CCB nimodipine (9). Interestingly, the protective effect of nimodipine occurs without altering proximal vasospasm; thus, the effect of this CCB-based treatment is thought to occur on the microvascular level (10). Beyond prophylactic nimodipine, once vasospasm or DCI develops, there is no proven treatment to address them.

Recent data suggest that the intrathecal (IT) delivery of the CCB nicardipine may have a role in a reactive (rather than a preventative) treatment approach to address cerebral vasospasm and avoid DCI (11). This approach has the advantage of avoiding deleterious systemic effects, i.e., decreased blood pressure, that is common with oral and intravenous CCBs (12). An intermittent and titratable IT nicardipine regimen was shown to be associated with proximal vessel vasodilation, reduced risk for DCI, and improved long-term functional outcomes (11, 13). However, the macrovascular vasodilation itself, as quantified by a reduction in daily TCD blood flow velocity, was not associated with outcomes (11). Therefore, the underlying mechanism driving the effect of IT nicardipine on patient outcomes remains unclear.

Both DCI and, to a more controversial extent, vasospasm is thought to occur on the microvascular level (13–16). However, the efficacy of IT nicardipine has only been assessed to date in humans at the macrovascular level (11, 13, 17–19). Although the arteriolar tone and capillary diameter are governed by calcium-dependent processes (the latter *via* pericytes regulating capillary size) (20, 21), IT delivery does not guarantee that nicardipine successfully diffuses out of the subarachnoid space and reaches the smaller blood vessels within the brain tissue (20, 21). Further investigation into the influence of IT nicardipine on the microvasculature is needed to complement the existing body of work on its macrovascular effects (11, 13) and to improve our mechanistic understanding of the treatment's effect.

Current neuroimaging modalities capable of characterizing the microvascular hemodynamic response to IT nicardipine are limited in the critical care environment. While perfusion magnetic resonance imaging (MRI), positron emission tomography (PET), and/or computed tomography (CT) can quantify microvascular CBF, the cost, need for patient transport, and a lack of continuous monitoring limit their feasibility for capturing transient hemodynamic changes at the bedside. Brain tissue oxygen monitors (e.g., Licox^®^) have the inherent disadvantage of their invasive nature. Other indirect measures of microvascular flow, like non-invasive near-infrared spectroscopy measurements of hemoglobin oxygen saturation, are poor surrogates of CBF (22).

In this study, we employ a non-invasive, well-validated optical technique known as diffuse correlation spectroscopy (DCS) to measure regional microvascular CBF (23). In DCS, a near-infrared light source is used to illuminate the tissue, and a detector is placed some distance away to collect light that has randomly scattered throughout the underlying tissue. Light scattering of moving red blood cells leads to temporal fluctuations in detected light intensity. These temporal fluctuations are quantified through a normalized temporal intensity autocorrelation function, g_2_ (τ), that is fit to simple analytical models, to extract a blood flow index (BFI) (23). Although the units of BFI (cm^2^/s) differ from the traditional units of blood flow (ml/min/100 g), numerous validation studies demonstrate that BFI correlates with CBF measured by other “gold-standard” modalities (e.g., MRI) (24, 25).

Herein, we use DCS to test the hypothesis that IT nicardipine increases microvascular CBF from pre-administration levels in the absence of systemic changes. We also explore factors that are associated with the microvascular flow response, including patient characteristics, systemic hemodynamic response, baseline macrovascular flow velocities, and the development of DCI. Finally, we determine the correlation between microvascular CBF measured by DCS and macrovascular flow velocities measured by transcranial Doppler ultrasound (TCD).

## 2. Methods

### 2.1. Study design

Adult patients with non-traumatic SAH in the neurocritical care unit at Emory University Hospital (Atlanta, GA, USA) who had an external ventricular drain (EVD) placed as part of their usual care were enrolled. This observational study was approved by the Emory University Institutional Review Board (IRB00107474). Written and informed consent was obtained prior to study initiation from all patients or their surrogates.

Once consented, patients were included for monitoring only if (1) they developed large-artery vasospasm and (2) they were deemed to require IT nicardipine by the treating clinical teams (neurocritical care and vascular neurosurgery) as part of their usual care. Diagnosis of large-artery vasospasm was confirmed by either TCD, CT angiography, or digital subtraction angiography, according to accepted definitions (26). TCD was performed daily, while CT angiography was performed in patients without sonographic bone windows on a less frequent basis. Digital subtraction angiography was mostly performed in response to a suspected change in the exam that could be related to macrovascular vasospasm.

Patients received 4–5 mg of nicardipine mixed with 2 ml of NaCl 0.9% intrathecally through an existing EVD. After administration, the EVD was clamped for up to 30 min to promote the uptake of nicardipine by the local tissue microenvironment. Treatment dose, frequency, and length were determined by the attending physician and were not influenced by study enrollment.

Patient outcome was defined by the presence or absence of DCI on the recent imaging available within 6 weeks from admission, according to the consensus criteria (27). Specifically, imaging was performed 24–48 h after aneurysm occlusion in all patients as part of their usual care. DCI was defined as a new infarct noted on follow-up imaging that was not present on the post-occlusion scan and that did not have another explanation (e.g., brain compression from cerebral edema, EVD-related infarction, etc.). Follow-up imaging was performed periodically at the discretion of the attending physician, typically every 2–4 days during the ICU stay. All available imaging studies (CT and MRI) were reviewed by at least two board-certified neurocritical care attending physicians (OS, PK, and FA), who were blinded to the DCS observed blood flow changes, and a consensus decision was achieved regarding the presence of DCI.

### 2.2. Measurements of cerebral blood flow

To quantify the cerebral hemodynamic effects of IT nicardipine, continuous measurements of microvascular CBF were made with DCS during the first dose of IT nicardipine treatment.

DCS measurements were made with a custom-built instrument comprised of an 852-nm-long coherence laser (iBEAM smart, TOPTICA Photonics, Farmington, NY), eight photon-counting avalanche photodiodes (SPCM-AQ4C-IO, Perkin-Elmer, Quebec, Canada), and a counter/timer data acquisition board (PCIe-6612, National Instruments) (28). Detected photon counts were used to estimate an intensity autocorrelation function, g_2_ (τ), with a custom software correlator (28) (LabVIEW, National Instruments).

**Figure 1:**
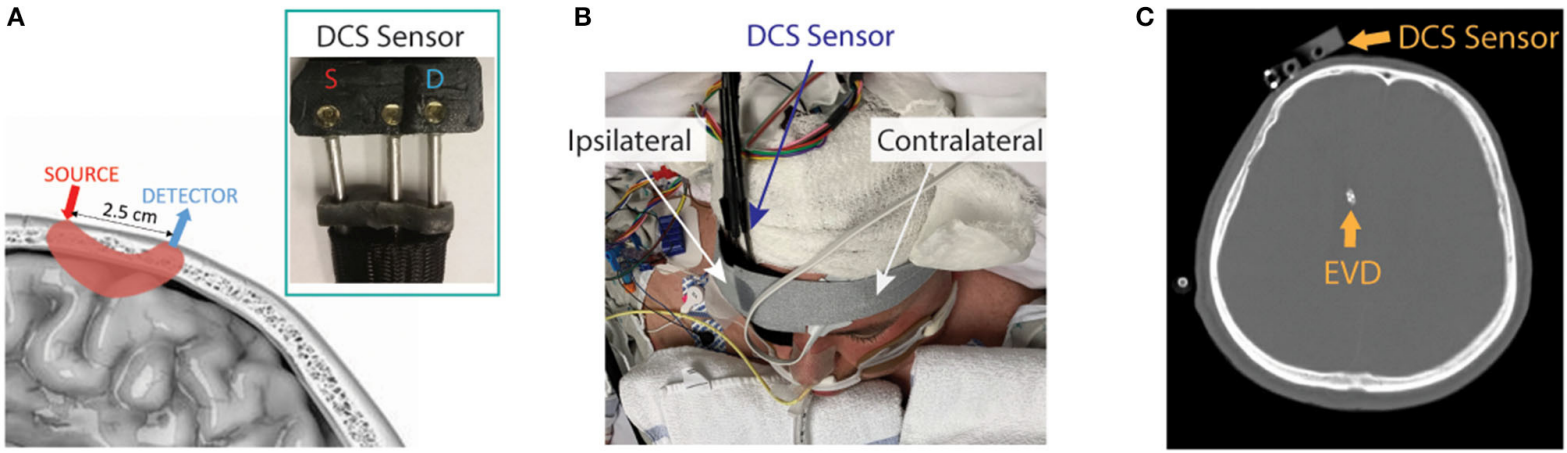
Diffuse correlation spectroscopy (DCS) experimental setup. **(A)** Pictorial representation of the DCS sensor on the scalp and the optical interrogation path for the source and detector geometry (pink shaded region), along with a photograph of the sensor (inset). **(B)** The DCS sensor was secured to the patient's forehead (patient image obtained with specific consent). **(C)** Representative CT scan showing typical placement of the DCS sensor and external ventricular drain (EVD).

The patient interface for these measurements consisted of a custom-made, flexible sensor containing a 1-mm fiber bundle source (FTIIG23767, Fiberoptics Technology, Pomfret, CT) spaced 2.5 cm from a bundle of seven 4.4 μm single-mode detection fibers (780HP, Thorlabs, Newton, NJ) (Figure 1A). All fibers were coupled to right-angle mirrors (MRA05-E03, Thorlabs, Newton, NJ) to allow them to lie flush with the forehead. The sensor was placed on the forehead of the hemisphere with the higher MCA velocities on TCD, suggesting poor macrovascular vasospasm. It was secured with medical tape (3M^TM^ Durapore Surgical Tape) and a head strap (Figures 1B, C). If large-artery vasospasm was observed in both hemispheres, the sensor was placed on the hemisphere with higher detected light intensities to maximize the signal-to-noise ratio (SNR). The sensor was positioned up to 5 min prior to IT nicardipine initiation, and monitoring at 0.3 Hz ensued for up to 90 min post-administration. Monitoring was discontinued prior to 90 min if the patient received secondary vasodilators (e.g., oral nimodipine and intravenous milrinone) or if the sensor had to be removed due to a clinical event (e.g., vomiting). The entire dataset was discarded if monitoring was discontinued <15 min after nicardipine administration.

To determine the effect of nicardipine on microvascular CBF, the measured intensity autocorrelation functions, *g*_2_(*t*, τ), at time, t, and delay time, τ, were fit for a blood flow index [BFI(t)] using the semi-infinite solution to the correlation diffusion equation (23). For these fits, we assumed a constant absorption coefficient, reduced scattering coefficient, and tissue index of refraction of 0.2 cm^−1^, 10 cm^−1^, and 1.4, respectively (29). Fits were constrained to *g*_2_(*t*, τ)>1.2 to increase sensitivity to the cerebral layer (30). Data were excluded if the detected light intensity was < 8 kHz due to a poor SNR. Finally, the relative change in cerebral blood flow (rCBF) as a function of time was calculated as follows:


(1) $$\begin{array}{l} rCBF\left(t\right)=\frac{BFI\left(t\right)-BFI_{baseline}}{BFI_{baseline}}\times 100\% \end{array}$$

where *BFI*_*baseline*_ is the temporal mean of *BFI(t)* over the 5-min baseline immediately prior to IT nicardipine administration.

### 2.3. Measurements of blood pressure, heart rate, and intracranial pressure

To investigate the potential confounding systemic influences of blood pressure and heart rate (HR), as well as any influence of intracranial pressure (ICP), on the cerebral hemodynamic response to IT nicardipine, we simultaneously captured blood pressure, HR, and ICP. As per standard clinical care, continuous arterial blood pressure was measured with a transducer in the radial artery leveled at the heart. Blood pressure was captured at 125 Hz with an analog-to-digital converter board (NI USB-6343) beginning 5 min before the first dose of IT nicardipine and continuing for up to 90 min post-administration. These data were time-synced with DCS measurements of CBF. Beat-to-beat values of systolic, diastolic, and mean blood pressure (MAP), as well as HR, were derived from the arterial blood pressure waveform in post-processing using the *delineator* function (31) in MATLAB (MathWorks, Natick, MA).

Intracranial pressure was measured with a transducer in the EVD (INS8401, Integra, Plainsboro, NJ). In general, the EVD was opened to allow for spontaneous CSF drainage; thus, continuous ICP measurements were not available. However, the EVD was clamped for the first 30 min after the administration of IT nicardipine, as well as briefly before and after this 30-min window. Thus, ICP was stochastically captured by hand every 1–15 min for up to 90 min post-administration.

To quantify the effect of IT nicardipine on HR, MAP, and ICP, we estimated the change in each parameter from pre-administration levels, e.g., Δ*MAP*(*t*) = *MAP*(*t*)−*MAP*_*baseline*_, where *MAP*_*baseline*_ is the temporal mean of *MAP(t)* over the 5-min baseline immediately prior to IT nicardipine administration.

### 2.4. Measurement of macrovascular blood flow velocity

To investigate the relationship between microvascular and macrovascular perfusion, we compared the average DCS-measured BFI in 5 min prior to IT nicardipine administration to TCD measures of mean CBF velocity in the middle cerebral artery (MCA) and anterior cerebral artery (ACA). We focused our investigation on these vessels because our DCS sensor was positioned near the intersection of the MCA and ACA vascular territories (Figure 1). TCD data were acquired as per usual clinical care on the morning of the study, prior to IT nicardipine administration (Dolphin IQ, Viasonix, TN) by a single operator. For each vessel, the maximum value of the time-averaged mean velocities (32) on the hemisphere of DCS monitoring was used to compare with DCS.

### 2.5. Statistical analysis

To test our hypothesis that IT nicardipine increases microvascular CBF in the absence of systemic changes, we first graphically explored rCBF, MAP, HR, and ICP as a function of time after nicardipine administration. Data were downsampled to 10-min intervals to aid in visualization. Next, a linear mixed-effects model was applied to the downsampled data to determine significant trends in each measured variable with time, where we accounted for repeated measures within participants (rCBF ~ time, fixed intercept). This model satisfies the assumptions of normality. For the outcomes MAP, HR, and ICP, we modeled subject-specific intercepts and slopes. For outcomes that were relative to time zero (e.g., rCBF, Δ MAP), we modeled subject-specific slopes. The model R^2^ was used to determine the goodness of fit. Furthermore, because we observed a heterogeneous response of rCBF to nicardipine, we used a latent class mixture model (33) (LCMM) to classify CBF responses into unobserved groups (i.e., latent classes) with similar, more homogenous patterns of rCBF. In this model, time post-nicardipine initiation was treated as the linear fixed effect, and the number of latent classes (e.g., distinct rCBF trajectories) was specified *a priori*. Given our relatively small sample size, we tested both 2 and 3 latent class solutions. No differences in the log-likelihood statistics were observed between the 2- vs. 3-class solutions; therefore, we employed a 2-class solution. Patients were assigned to a latent class (e.g., response pattern group) based on the class with the highest posterior probability of class membership. We used chi-square and Wilcoxon rank sum tests to determine the differences between these empirically derived classes in age, sex, SAH grade, time to treatment initiation, the total number of IT nicardipine doses, intracranial and mean arterial pressure pre-administration, TCD velocities pre-administration, and incidence of DCI.

To determine the relationship between microvascular CBF measured with DCS and macrovascular CBF velocity measured with TCD, Spearman's rank coefficient, *r*_*s*_, was used to assess the extent to which a linear model explains the variability in the relationship between BFI_baseline_ and mean blood flow velocities in the MCA and ACA.

Data are reported as mean ± standard deviation unless otherwise stated. LCMM analysis was performed with the LCMM package in R (version 3.5.2, rproject.org); all other analyses were performed in MATLAB. Statistical significance was assessed at the 0.05 level.

## 3. Results

Between 1 April 2019 and 31 October 2021, 57 patients with non-traumatic SAH (aneurysmal or diffuse pattern angiogram negative) admitted to the neurocritical care unit at Emory University Hospital consented. Of 57 patients, 37 developed clinically significant large-artery vasospasm that required treatment with IT nicardipine, as determined by the treating-attending physician. The CBF response to the first dose of IT nicardipine was monitored in 27 out of 37; the remaining 10 were excluded due to the availability of the research-based DCS device. Of those 27 monitored with DCS, 20 (74%) were included in our final analysis; the remaining 7 datasets were excluded due to measurement duration of <15 min. Of these seven discarded datasets, premature discontinuation was caused by patient discomfort related to IT administration and EVD clamping (*n* = 3), failure to properly secure the DCS sensor on the forehead (*n* = 3), or co-administration of oral nimodipine at the time of DCS monitoring that induced confounding effects on CBF (*n* = 1). The average duration of DCS monitoring post-nicardipine was 82.7 ± 23.2 min.

Patients were mostly female (70%), with a mean ± standard deviation age of 48.5 ± 10.1 years (Table 1). Aneurysm location and grade severity were heterogeneous, as described in Table 2. An IT nicardipine dose of 4 or 5 mg was administered every 6–8 h with an average of 36 ± 20 doses. Treatment was initiated on post-bleed day 5 ± 1. One patient had evidence of DCI on CT prior to nicardipine initiation. Five out of 20 developed new DCI on the recent imaging available (up to 6 weeks post-bleed); the mean time between the first dose of IT nicardipine to DCI development was 5 ± 4 days. Supplementary Figure 1 shows the DCI-related infarction of each patient diagnosed with DCI.

**Table 1:** Summary of patient cohort.

**Demographic information and risk factors**	
Age (y)	48.5 ± 10.1
Sex—female, *N* (%)	14 (70%)
**Race**	
African American	11 (55%)
White	8 (40%)
Asian	1 (5%)
Hispanic	2 (10%)
Hypertension, N (%)	12 (60%)
Coronary artery disease, *N* (%)	1 (5%)
Diabetes mellitus, *N* (%)	1 (5%)
Tobacco use, *N* (%)	11 (55%)
Dyslipidemia, *N* (%)	2 (10%)
Depth to brain (cm)	1.2 ± 0.3
Total IT nicardipine doses (N)	36 ± 20

Data are reported as mean ± standard deviation or N (%).

**Table 2:** Summary of patient demographics and clinical characteristics.

**Sex**	**Age**	**WFNS**	**Aneurysm location**	**Aneurysm treatment**	**Sensor location**	**DCI**	**DCI location**
F	61	4	R PCom	Endovascular	L Frontal	No	
F	47	4	R Ant Choroidal	Clip ligation	R Frontal	Yes	R MCA
F	62	5	Angio Negative		L Frontal	Yes	R MCA
F	62	2	L PCom	Clip ligation	L Frontal	No	
F	37	4	R Vertebral	Endovascular	R Frontal	No	
M	44	5	R PICA	Endovascular	R Frontal	No	
F	49	2	R PCom	Clip ligation	R Frontal	No	
M	56	2	L PCom	Endovascular	L Frontal	Yes	L MCA
F	58	4	ACom	Endovascular	R Frontal	Yes	L ACA
M	33	1	L MCA	Endovascular	L Frontal	No	
F	35	2	L MCA	Endovascular	L Frontal	Yes	L and R ACA
M	50	4	L SCA	Clip ligation	L Frontal	No	
F	58^*^	5	R MCA	Clip ligation	L Frontal	Yes	R SCA
M	43	4	ACom	Endovascular	L Frontal	No	
F	52	2	ACom	Endovascular	R Frontal	No	
M	37	2	ACom	Endovascular	L Frontal	No	
F	64	2	R MCA	Endovascular	R Frontal	No	
F	44	4	L PCom	Endovascular	L Frontal	No	
F	38	5	L ICA	Endovascular	R Frontal	No	
F	40	2	L MCA	Clip ligation	L Frontal	No	

Sex: M, male; F, female; SAH grade at admission: WFNS, World Federation of Neurosurgical Societies grade; DCI, delayed cerebral ischemia; R, right; L, left; PCom, posterior communicating artery; Ant, Anterior; PICA, posterior inferior cerebellar artery; ACom, anterior communicating artery; MCA, middle cerebral artery; SCA, superior cerebellar artery; ICA, internal carotid artery.

DCI was determined based on the recent imaging available (up to 6 weeks). ^*^Denotes DCI developed prior to treatment.

**Figure 2:**
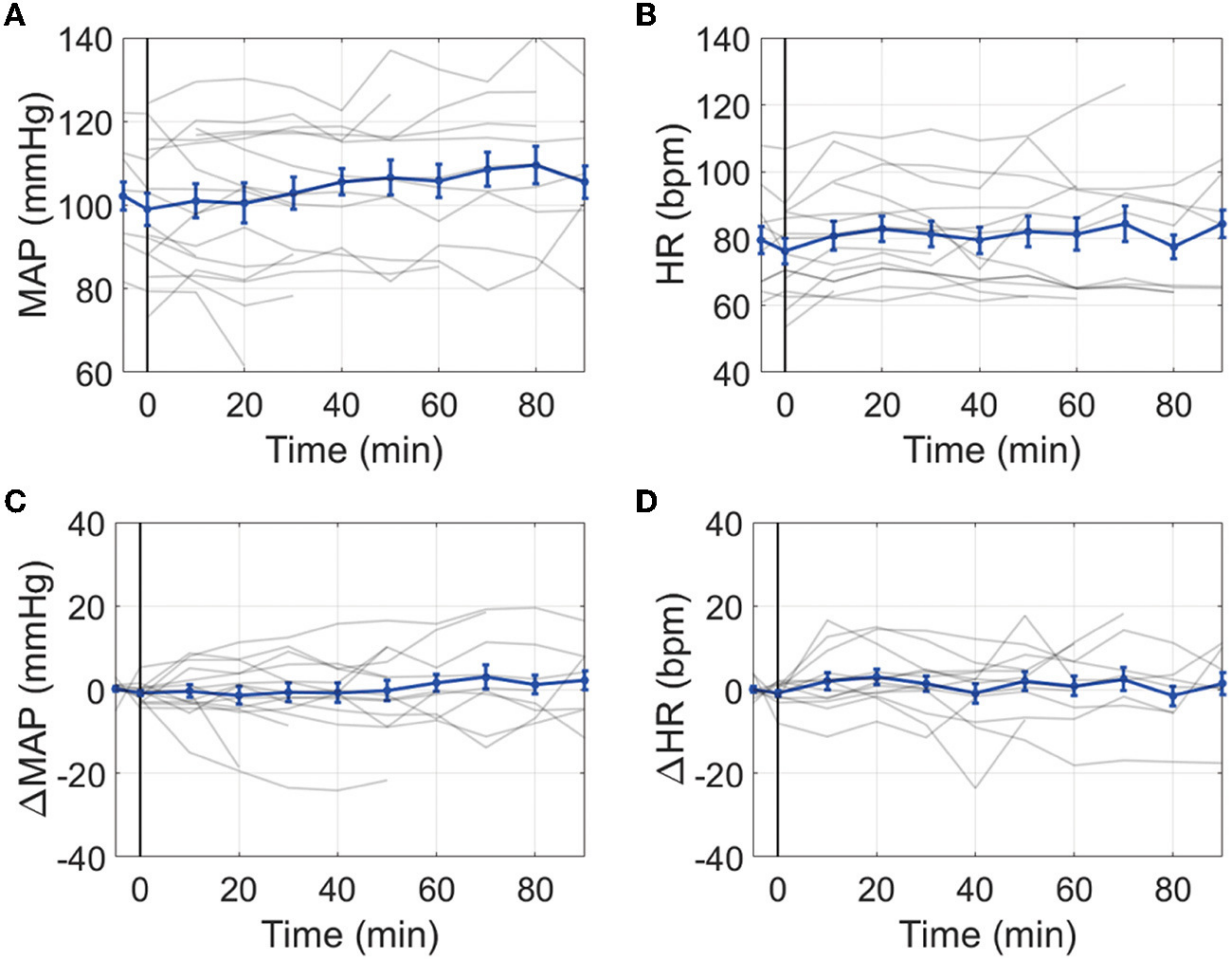
Effect of IT nicardipine on systemic blood pressure and heart rate: Mean arterial pressure [MAP, **(A)**], heart rate [HR, **(B)**], change in MAP from pre-administration levels [ΔMAP, **(C)**], and change in HR from pre-administration levels [ΔHR, **(D)**] as a function of time post-administration of IT nicardipine. Thin gray lines in each subplot denote individual patient responses, and thick blue lines denote the mean and standard deviation across all patients. The black vertical line at time = 0 denotes the start of treatment.

The average MAP prior to IT nicardipine administration was 103 ± 16 mmHg; the average HR was 80 ± 14 bpm. After administration, no significant trends with time were observed for MAP or HR (both *p* > 0.05, Figure 2). Average ICP at the start of IT nicardipine and 30 min post-administration was 9 ± 4 mmHg and 11 ± 5 mmHg, respectively (*p* > 0.05); ICP remained unchanged from IT nicardipine administration during the monitoring session (*p* = 0.8, data not shown).

**Figure 3:**
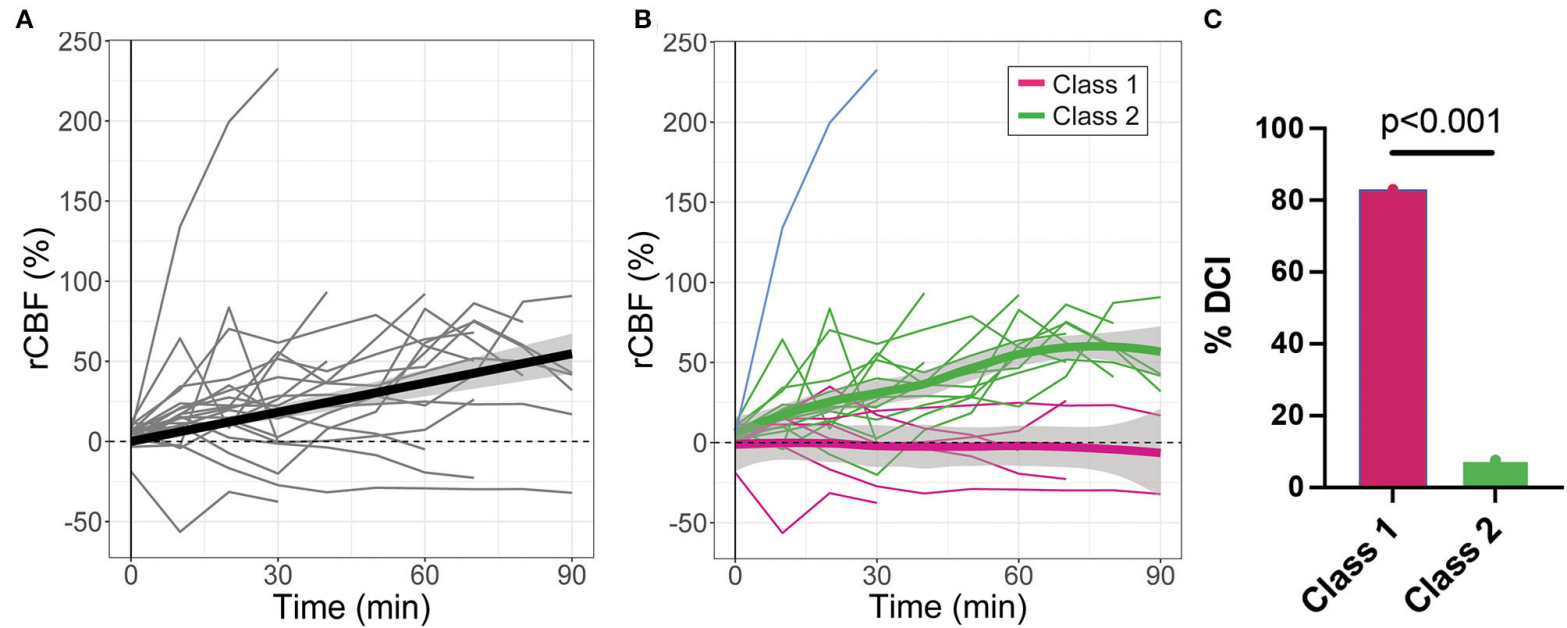
Relative changes in cerebral blood flow (rCBF) as a function of time post-IT nicardipine administration. **(A)** Individual patient rCBF trajectories (gray) along with the linear mixed-effects model fit (black). **(B)** Two distinct latent classes of rCBF response were identified by the latent class mixture model: Class 1 (n = 6, in magenta) showed minimal change in rCBF post-nicardipine, whereas Class 2 (n = 13, in green) exhibited an increase in rCBF over the course of monitoring. The model was overly sensitive to one distinct trajectory (in blue); this outlier trajectory was excluded from analysis. Here, thin lines denote individual patient responses, while thick lines denote the model best fit for each response class. **(C)** Bar plot of the indicidence of delayed cerebral ischemia (DCI) by rCBF response class.

Microvascular CBF measured with DCS significantly increased with time after IT nicardipine administration (slope = 0.64%/min, SE = 0.09, *p* < 0.001), although appreciable response heterogeneity was noted across subjects (Figure 3A). Using a latent class mixture model, 19 out of 20 patients were classified into two distinct classes of CBF response; Class 1 (*n* = 6, in magenta in Figure 3B), on average, showed a minimal change in rCBF post-administration, whereas Class 2 (*n* = 13, in green in Figure 3B), on average, exhibited a steady increase in rCBF over the course of monitoring. Notably, for this analysis, visual inspection revealed 1 distinct trajectory that the LCMM model was overly sensitive (shown in blue in Figure 3B); this outlier trajectory was excluded from the LCMM analysis. No significant differences in age, SAH grade, number of doses, depth to the brain, baseline MAP, baseline HR, or baseline ICP were seen between the LCMM-identified classes of CBF response to nicardipine (Table 3, all *p* > 0.05). Patients in Class 1 were mostly female (100% vs. 54% in Class 2, *p* = 0.044) and tended to have a higher pretreatment MCA velocity in the hemisphere of DCI monitoring (151 vs. 126 cm/s in Class 2, *p* = 0.02). Most strikingly, the frequency of DCI was significantly different between classes (*p* < 0.001); 5 out of 6 subjects in Class 1 had DCI, 4 of whom developed new DCI, despite treatment with IT nicardipine, on the recent imaging available up to 6 weeks post-bleed, and 1 of whom started with DCI prior to nicardipine, while only 1 out of 13 subjects in Class 2 had DCI (Figure 3C). Further review of the excluded outlier revealed that the CBF trajectory was correlated with these trends; this patient had a sharp increase in CBF, similar to those in Class 2, and did not develop DCI.

**Table 3:** Summary of patient differences between cerebral blood flow response classes.

	**Class 1 (*n =* 6)**	**Class 2 (*n =* 13)**	***p*** **-value**
Age (y)	52 (38, 58)	44 (39, 57)	0.783
Sex—female N (%)	6 (100)	7 (54)	0.044
WFNS	4 (4, 5)	2 (2, 4)	0.176
Depth to brain (mm)	11.2 (9.4, 13.0)	12.3 (9.1, 14.6)	0.831
MAP (mmHg)	99 (87, 114)	101 (92, 112)	0.594
ICP (mmHg)	12 (8, 13)	7 (4, 13)	0.374
MCA velocity (cm/s)	151 (142, 179)	126 (106, 140)	0.020
ACA velocity (cm/s)	99 (77, 133)	100 (79, 118)	0.941
BFI (1 × 10^8^ cm^2^/s)	1.0 (0.7, 1.5)	1.0 (0.7, 1.4)	0.898
Number of doses of IT nicardipine	50 (36, 67)	34 (13, 46)	0.085
DCI—*N* (%)	5 (83)	1 (8)	9.768e-4

Data are reported as median (IQR) or N(%). Baseline mean arterial pressure (MAP), intracranial pressure (ICP), and DCS-measured blood flow index (BFI) were assessed in 5-min period before the first dose of intrathecal nicardipine was administered. Maximal time-averaged mean TCD velocities in the middle cerebral artery (MCA) and anterior cerebral arteries (ACA) from the hemisphere of DCS monitoring were assessed on the morning of IT nicardipine administration. P-values were assessed with chi-square and Wilcoxon rank sum tests for categorical and continuous data, respectively.

**Figure 4:**
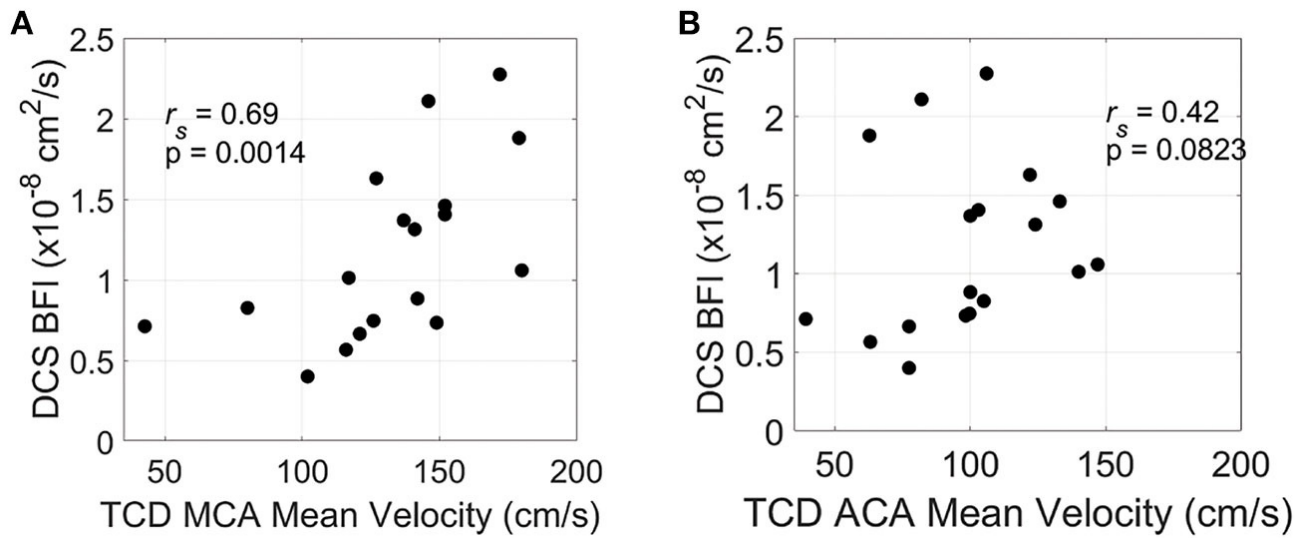
Relationship between resting-state and pretreatment micro- and macrovascular blood flow. Mean blood flow velocity in the middle cerebral artery [MCA, **(A)**] and anterior cerebral artery [ACA, **(B)**] measured by transcranial Doppler ultrasound (TCD) on the hemisphere of DCS monitoring vs. diffuse correlation spectroscopy (DCS)-measured blood flow index (BFI). TCD and DCS data were both acquired prior to IT nicardipine administration.

Of 20 patients, 18 had TCD measurements prior to IT nicardipine initiation (4.5 ± 1.9 h, time difference between TCD and DCS measurements). Average pretreatment MCA and ACA maximal mean blood flow velocities were 132.3 ± 34.2 cm/s and 98.9 ± 28.5 cm/s, respectively. In this subset of patients, we observed a significant positive correlation between mean resting-state blood flow velocity in the MCA and baseline, pretreatment BFI measured by DCS (*r*_*s*_ = 0.69, *p* = 0.0014) (Figure 4A). A similar trend was seen between mean flow velocity in the ACA and baseline BFI, although it did not reach statistical significance (*r*_*s*_ = 0.42, *p* = 0.083, Figure 4B).

## 4. Discussion

Here, we used DCS to quantify the microvascular CBF response to IT nicardipine administration in SAH patients with large-artery cerebral vasospasm. On average, microvascular CBF increased over 90 min of monitoring. This CBF increase was observed in the absence of systemic changes in blood pressure or HR, suggesting that the cerebral hemodynamic response is likely due to a local drug effect rather than systemic influences and further strengthening the plausibility of microvascular effects on top of the macrovascular effects, as previously reported (11, 13). However, we observed substantial heterogeneity in this response across patients. A latent class mixture model was used to objectively identify two distinct classes of CBF response, i.e., Class 1, wherein a minimal-to-no change in CBF was observed, and Class 2, wherein a steady increase in CBF was observed. Interestingly, this differential CBF response was significantly associated with DCI. Notably, 1 out of 13 patients with increased CBF after IT nicardipine developed DCI, while 5 out of 6 patients whose CBF did not respond to IT nicardipine had DCI. Although these are preliminary observations, they suggest that the CBF response to the first dose of IT nicardipine could be a novel biomarker for treatment response and DCI risk, alerting clinicians to consider alternative interventions. Given the strong association between DCI and poor functional outcome (4, 34, 35), real-time identification of high-risk patients, or those who do not respond to nicardipine treatment, is of high clinical significance.

Several possible mechanisms may explain the lack of CBF response to nicardipine in the Class 1 subset. MCA velocity was higher in Class 1 compared to Class 2 (*p* = 0.02, Table 3), suggesting that the severity of MCA vasospasm may be worse in Class 1. Many vasospasm-independent mechanisms, including microthrombosis, venous outflow obstruction, and cortical spreading depolarization, may be present in these patients that are unaffected by nicardipine (36, 37). Alternatively, the additional volume of drug and diluent into the cranial vault could potentially transiently decrease cerebral perfusion, while ICP remains minimally changed. Furthermore, the timing of treatment may influence the CBF response. Using a rabbit SAH model, Vorkapic et al. (38) demonstrated two phases of large-artery vasospasms, i.e., an early pharmacologically reversible phase and a later irreversible phase. During the acute reversible vasospasm stage, cerebral vasoconstriction could be reversed by CCBs. However, as organic changes such as intimal proliferation, edema, and degeneration occur, the efficacy of pharmacological interventions is reduced (17, 38). Regardless of the mechanism, coupling DCS with a pharmacological challenge may be a valuable risk-stratification tool to measure a treatment effect in the microvascular bed alongside TCD assessment of the macrovasculature to better guide clinical decisions and minimize DCI after SAH.

A secondary aim of this study was to investigate the relationship between DCS measurements of resting-state, pretreatment microvascular blood flow, and TCD measurements of macrovascular flow velocity. We observed a moderate positive correlation between the DCS-measured BFI which is measured prior to nicardipine administration and the TCD-measured mean blood flow velocity in the MCA which is measured on the morning of DCS monitoring. A similar correlation was trending toward significance in the ACA (*p* = 0.082). Of note, the sensor was positioned over the watershed area between the ACA and MCA territories. This positive correlation between resting-state microvascular and macrovascular flow is seen in healthy adults, including by DCS vs. TCD (39, 40), as well as in brain-injured cohorts, including SAH (41–43). In SAH patients with macrovascular vasospasm, we expected a negative or null correlation, as elevated TCD velocities are indicative of vasoconstriction that may have downstream effects of reducing microvascular blood flow. However, microvascular blood flow is reported to only be significantly reduced in the setting of severe large-artery vasospasm (44), while most of our cohort was classified as either mild or moderate vasospasm. These results may suggest that this macrovascular and microvascular correlation in the MCA territory remains intact at rest in our cohort of patients with mild-to-moderate large-artery vasospasm. We note that TCD and DCS were not measured concurrently during/post-administration of IT nicardipine due to technical challenges of simultaneous monitoring; thus, we cannot infer how the treatment influences macro-micro correlation. In the future, measurements of microvascular blood flow with DCS in concert with macrovascular TCD assessments may serve a role in dissecting the complicated CBF dynamics after SAH.

Our study has several limitations. First, by design, this was an observational study that involved dynamic monitoring of patients treated as part of their usual care without a placebo comparison. Limitations regarding the small sample size and study design necessitate confirmation of these results in the setting of a randomized controlled trial. Second, seven (26%) DCS monitoring sessions were discarded from the analysis. While this incidence is appreciable, we anticipate a significantly lower discard rate with continued future enrollment as we continually make improvements to the research device that enhances SNR. Third, based on previous reports of a short half-life of nicardipine (18, 45), we limited monitoring for up to 90 min, although monitoring was discontinued prior to 90 min in several patients due to instrument availability or the administration of therapeutic agents known to exert confounding influences on cerebral hemodynamics (e.g., oral nimodipine). With a larger sample size and extended monitoring, we will explore the necessary duration of monitoring required to robustly distinguish the dichotomy between the patients who did and did not respond favorably to IT nicardipine. Fourth, we were limited by SNR to a single measurement location for DCS, which we selected to be the hemisphere in which higher TCD velocities were measured (suggesting worse large-artery vasospasm). As a result, it is difficult to ascertain if our local measurement is indicative of changes that persist throughout the brain, or if there is regional heterogeneity in the response. In general, intrathecally administered nicardipine is expected to distribute within the CSF compartment across the subarachnoid space where blood vessels travel. Thus, the vasodilatory effects should be diffused as nicardipine permeates blood vessels within the subarachnoid space. However, the regional response accessible to DCS could differ from that of other vascular territories. In the future, increasing the number of laser sources and/or detectors may enable simultaneous monitoring of multiple locations around the head to determine if regional response heterogeneity is present as well as to explore factors associated with response heterogeneity (e.g., SAH and/or vasospasm location/severity). Finally, we employed the semi-infinite solution to the correlation diffusion equation (23) to analyze DCS data, which models the head as a homogenous medium. While the correlation with TCD suggests that this approach provides a suitable first-order approximation, future studies should employ multi-layer approaches (46) that enhance sensitivity to cerebral perfusion and minimize contributions from extracerebral layers.

In summary, we use DCS to characterize the cerebral hemodynamic response to IT nicardipine treatment in patients with large-artery cerebral vasospasm after SAH. We observed the following two distinct classes of microvascular CBF response: (1) minimal-to-no change and (2) a steady increase. We found that 83% of patients whose CBF failed to respond to nicardipine developed DCI, while 8% of patients whose CBF increased developed DCI. These results suggest that the acute (< 90 min) DCS-measured CBF response to IT nicardipine is associated with intermediate-term (up to 3 weeks) development of DCI. If these findings are corroborated with future, larger-scale prospective, randomized control trials, they suggest that DCS, alongside TCD, may provide a valuable biomarker to better inform clinical decisions, diminish the development of DCI, and improve functional outcomes following SAH.

## Data availability statement

The raw data supporting the conclusions of this article will be made available by the authors, without undue reservation.

## Ethics statement

The studies involving human participants were reviewed and approved by Emory University Institutional Review Board (IRB00107474). The patients/participants provided their written informed consent to participate in this study.

## Author contributions

Data acquisition: ES, KC, AL, SL, and ZF. Data analysis: ES. Data interpretation, discussion, and writing—reviewing and editing: ES, CM, FA, OBS, PK, OS, and EB. Statistical analysis and writing—original draft preparation: ES, CM, OS, and EB. Supervision: OS and EB. All authors have read and agreed to the published version of the manuscript.
