# Supplementary material for: Microvascular cerebral blood flow response to intrathecal nicardipine is associated with delayed cerebral ischemia
Source: Front Neurol. 2023 Mar 17;14:1052232. doi: 10.3389/fneur.2023.1052232 (PMC10064128; doi:10.3389/fneur.2023.1052232)
Supplement: Supplementary file 1 [file Image_1.pdf]

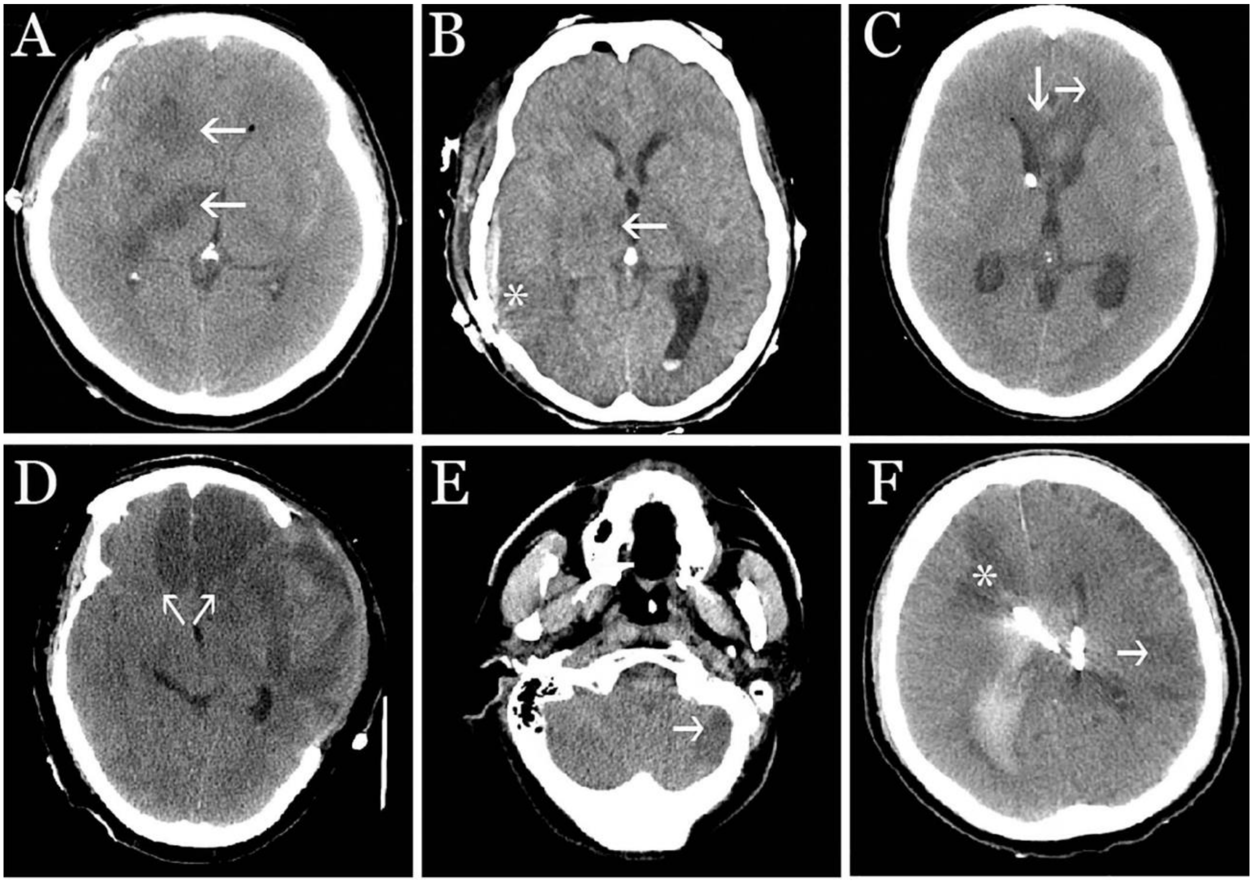

Supplementary Figure 1: CT images demonstrating infarct related to DCI identified in the presented cohort. (A–F) Examples from six different patients who were found to have DCI. White arrows denote the location of the infarction(s) related to DCI. *Denotes hypodense areas related to either post-surgical changes (B) or related to the external ventricular drain (F); such findings were not considered related to DCI.
